# Magnetic resonance imaging of the vagina: an overview for radiologists with emphasis on clinical decision making*

**DOI:** 10.1590/0100-3984.2013.1726

**Published:** 2015

**Authors:** Daian Miranda Ferreira, Régis Otaviano França Bezerra, Cinthia Denise Ortega, Roberto Blasbalg, Públio César Cavalcante Viana, Marcos Roberto de Menezes, Manoel de Souza Rocha

**Affiliations:** 1 MD, Resident at Service of Radiology, Instituto do Câncer do Estado de São Paulo Octavio Frias de Oliveira (Icesp), São Paulo, SP, Brazil.; 2 Physician Assistants at Service of Radiology, Instituto do Câncer do Estado de São Paulo Octavio Frias de Oliveira (Icesp), São Paulo, SP, Brazil.; 3 PhD, Physician Assistant at Instituto de Radiologia do Hospital das Clínicas da Faculdade de Medicina da Universidade de São Paulo (InRad/HC-FMUSP), São Paulo, SP, Brazil.; 4 PhD, Head of Unit of Radiology, Instituto do Câncer do Estado de São Paulo Octavio Frias de Oliveira (Icesp), São Paulo, SP, Brazil.; 5 Private Docent, Associate Professor, Department of Radiology, Faculdade de Medicina da Universidade de São Paulo (FMUSP), São Paulo, SP, Brazil.

**Keywords:** Magnetic resonance imaging, Vagina, Tumors, Congenital malformations

## Abstract

Magnetic resonance imaging is a method with high contrast resolution widely used in the assessment of pelvic gynecological diseases. However, the potential of such method to diagnose vaginal lesions is still underestimated, probably due to the scarce literature approaching the theme, the poor familiarity of radiologists with vaginal diseases, some of them relatively rare, and to the many peculiarities involved in the assessment of the vagina. Thus, the authors illustrate the role of magnetic resonance imaging in the evaluation of vaginal diseases and the main relevant findings to be considered in the clinical decision making process.

## Abstract (pt)

A ressonância magnética é um método com alta resolução de contraste e por isso muito utilizada na avaliação de doenças ginecológicas pélvicas. No entanto, seu potencial para diagnóstico de lesões vaginais ainda é subestimado, provavelmente em razão da escassa literatura referente ao tema, da pouca familiaridade dos radiologistas com doenças vaginais, algumas delas relativamente raras, e das muitas peculiaridades em um exame para avaliação desta víscera oca. Desta forma, ilustraremos neste estudo o papel da ressonância magnética na avaliação das doenças vaginais e os principais achados relevantes para a conduta clínica.

## INTRODUCTION

Vaginal lesions are detected at physical gynecological examination and, in a large portion of cases, the diagnosis is made by means of biopsy and anatomopathological analysis. Ultrasonography is utilized for complementary evaluation, but with a narrower scanning area and, consequently, limitation for locoregional staging. Computed tomography (CT) has poor contrast resolution and is limited to the diagnosis of pelvic lymph nodes in malignant diseases. Thus, over the past years, magnetic resonance imaging (MRI) has become the method of choice for the diagnosis of vaginal lesions, tumor staging, postoperative follow-up and treatment (chemotherapy and radiotherapy) response evaluation^(1,2)^.

MRI has gained ground in the evaluation of vaginal diseases due to its increasing availability and technological developments, which has allowed the development of faster and better quality protocols. Such protocols characterize the vaginal anatomy in detail, as well as its relationship with pelvic structures, besides allowing for a dynamic study during Valsalva maneuver, in the clinical suspicion of perineal descent. Additionally, diffusion and perfusion techniques have the potential to provide functional data to the traditional anatomical study.

Thus, the present study illustrates the role played by MRI in the evaluation of vaginal diseases, describing the main findings of relevance in the decision making about the clinical approach.

## METHOD

A 1.5 T MRI apparatus (General Electric; Milwalkee, USA) was utilized for images acquisition with T2-weighet fast spin echo (FSE) sequences, in the axial, sagittal and coronal planes, and T1-weighted gradient-echo (GRE) sequences. The diffusion technique was utilized with a high b value (~ 1.000 s/mm^2^) that is useful in the pre- and posttreatment evaluation of tumor lesions, as well as in the detection of lymph node involvement. Contrast-enhanced T1weighted sequences are routinely utilized in vagina-dedicated protocols; however, in some cases such images acquisition was not necessary. The use of aqueous gel is desirable and should be done whenever possible as it distends the vaginal cavity, allowing for a better evaluation of intraluminal lesions and parietal infiltration.

## ANATOMY

**Figure 1:**
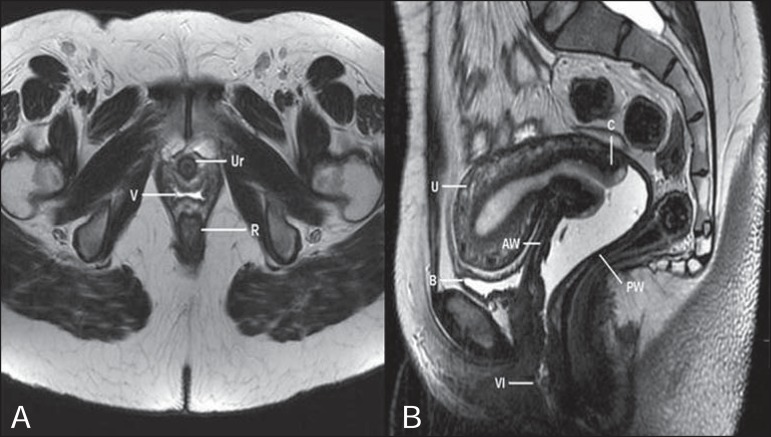
Normal female pelvis. Axial (**A**) and sagittal (**B**) MRI T2-weighted images showing anterior compartment containing the urethral ostium (Ur) and the ostium of the bladder (B), the medial compartment containing the uterus (U), the uterine cervix (C), the vagina distended with gel (V), the anterior wall of the vagina (AW), the posterior wall of the vagina (PW), the vaginal vestibule (VI) and the posterior compartment with the rectum (R).

The vagina is a median fibromuscular tubular structure that extends from the uterine cervix to the vulva, with an estimated length between 7 and 9 cm. whose wall consists of three layers as follows: mucosa, muscle and adventitia. Its anatomy is better studied at T2-weighted sequences, which can demonstrate the mucosa and intraluminal secretions with high signal intensity in contrast with the muscle layer, which presents with markedly low signal intensity (Figure 1).

## CONGENITAL ANOMALIES

### Transverse vaginal septum

It is a vertical fusion defect that occurs around the 20th gestational week, like other congenital vaginal malformations. It divides the vagina into two segments, reducing its functional length and causing obstruction of the vaginal canal.

**Figure 2:**
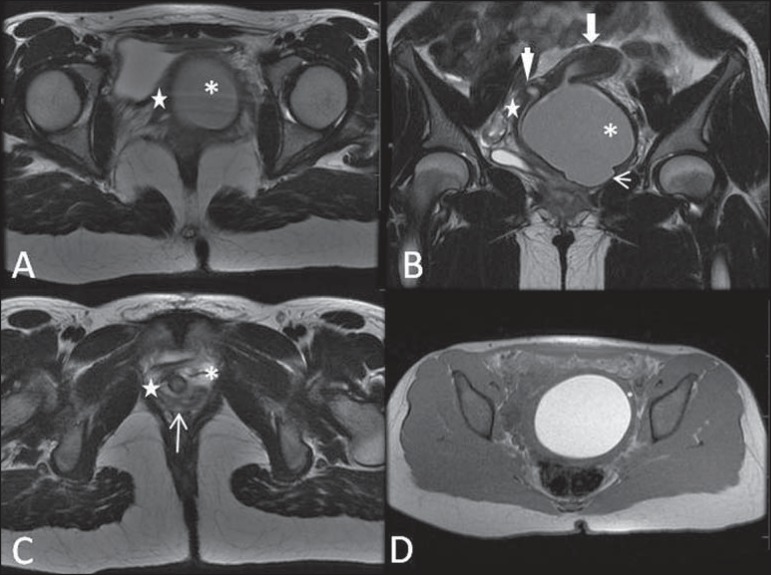
Uterus didelphys, longitudinal and transverse vaginal septa. MRI T2- weighted (**A,B,C**) and T1-weighted (**D**) sequences demonstrating longitudinal septum (thin arrow) dividing the vagina into two parallel cavities. The left hemivagina (asterisks) is obstructed by a transverse septum (thin arrowhead) and distended by hematic contents (high signal on T1- weighted image). Displaced and compressed right hemivagina at right (stars), right uterine horn (bold arrowhead), left uterine horn (bold arrow).

MRI is indicated for planning the septoplasty, as it evaluates the septum thickness and allows for the identification of the uterine cervix. It also can differentiate between upper vaginal septum and cervical agenesis, a relevant information to define the surgical approach^(2,3)^ (Figure 2).

### Longitudinal vaginal septum

**Figure 3:**
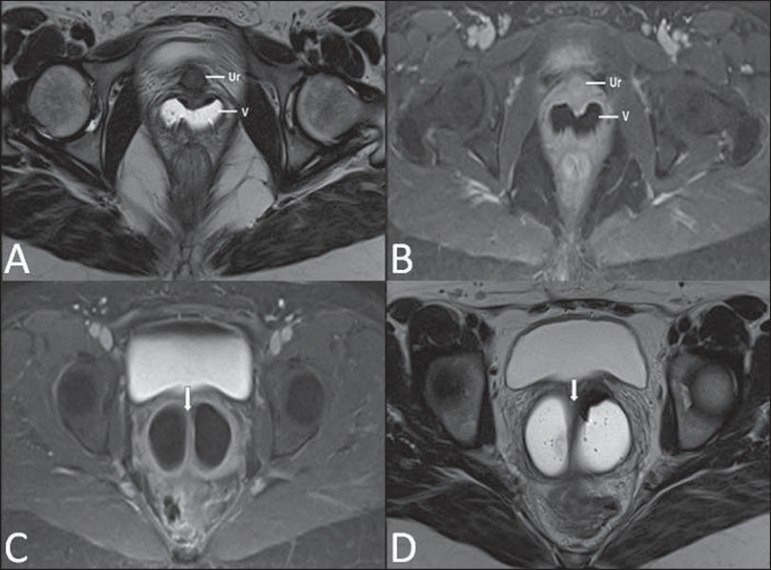
Longitudinal vaginal septum. Contrast-enhanced MRI T2- weighted (**A,D**) and T1-weighted (**B,C**) images showing longitudinal vaginal septum (arrows) dividing the vagina into two chambers. Ur, urethra; V, vagina. The identification at MRI may be difficult as the presence of the vaginal septum is not associated with obstruction.

Longitudinal vaginal septum is a lateral fusion defect of the Müllerian ducts, resulting in duplication of the uterus and vagina, in variable degrees^(2)^ (Figures 2 and 3).

### Imperforate hymen

**Figure 4:**
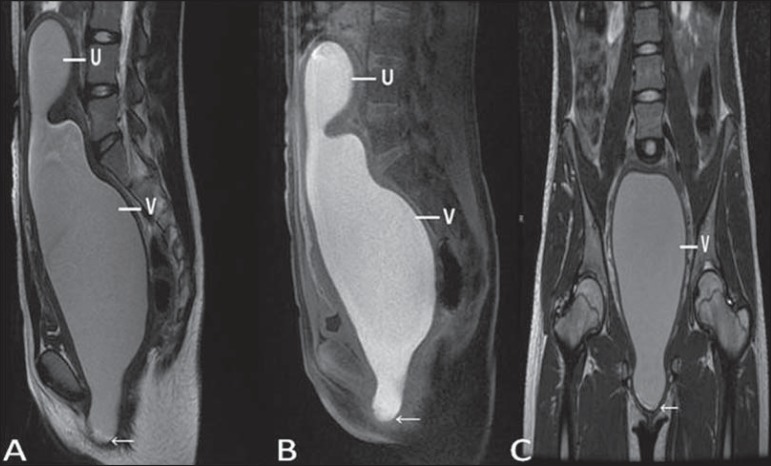
Imperforate hymen (arrow). MRI T2-weighted (**A**) and T1- weighted, sagittal (**B**) and coronal (**C**) sequences demonstrating uterus (U) and vagina (V) distended by hematic contents, which extends inferiorly protruding the ostium.

The hymen is a dermal membrane that wholly or partially occludes the external orifice of the vagina and is generally perforate. Imperforate hymen represents a failure in the vaginal recanalization process, and the diagnosis occurs mainly in the infancy by the bulging of the vaginal ostium caused by mucous secretion secondary to maternal estrogen stimulation, or during menarche^(2)^ (Figure 4).

### Androgen insensitivity syndrome

Androgen insensitivity syndrome determines failure in the development of the external genitalia in individuals with the 46,XY karyotype. Such syndrome results in decrease or absence of biological activity of androgens due to mutations in their receptor gene located in the X chromosome. Clinically, it may manifest as female phenotype with several degrees of virilization, secondary to partial or complete androgen insensitivity.

**Figure 5:**
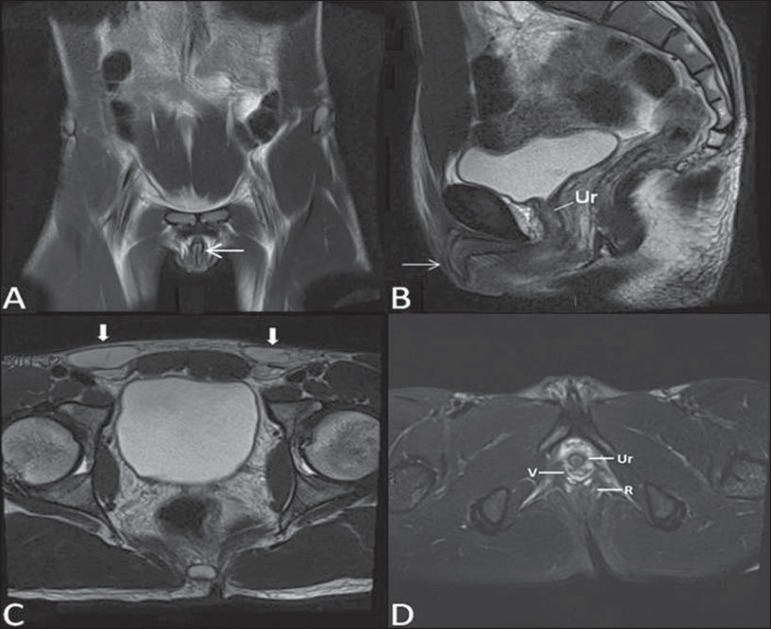
Partial androgen insensitivity syndrome. Multiplanar MRI T2- weighted sequences (**A,B,C**) and T2- weighted sequence with fat saturation (**D**) demonstrating masculine false hermaphroditism (46,XY) in a 21 years old patient with female phenotype and ambiguous genitalia, characterized by a short vagina (V) and presence of a micropenis (thin arrows). The images of the pelvis demonstrate neither uterus nor ovaries, and the testicles are located in the inguinal canals (bold arrows). Observe the hypertrophic rectoabdominal muscles, and the scarcity of subcutaneous fat caused by testosterone activity.

The diagnosis is usually made in puberty due to primary amenorrhea, and the testicles may be found in the inguinal canal, in the labia majora and in the abdomen^(4)^ (Figure 5).

### Mayer-Rokitansky-Kuster-Hauser syndrome

**Figure 6:**
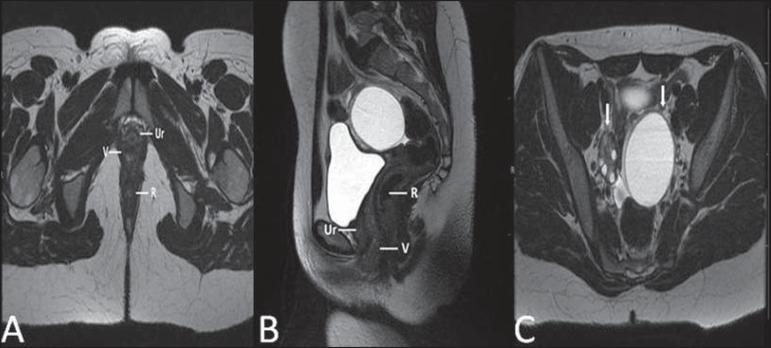
Mayer-Rokitansky-Kuster- Hauser syndrome (complete presentation). Multiplanar T2-weighted sequence (**A,B,C**) showing absence of the uterus and of the upper third of the vagina (V) between the rectum (R) and the urethra (Ur). The pelvic images confirm the presence of normal ovaries and large cystic mass in the left ovary.

It is a syndrome characterized by vaginal aplasia associated with other anomalies of the Müller ducts. The classical presentation consists in the absence of the uterus and of the proximal two thirds of the vagina, with variable degrees of compromising of these structures. Type I is characterized by isolated absence of the proximal two thirds of the vagina, while type II is characterized by the presence of other malformations such as vertebral, cardiac, urological and otological anomalies^(5)^ (Figure 6).

### Turner syndrome

**Figure 7:**
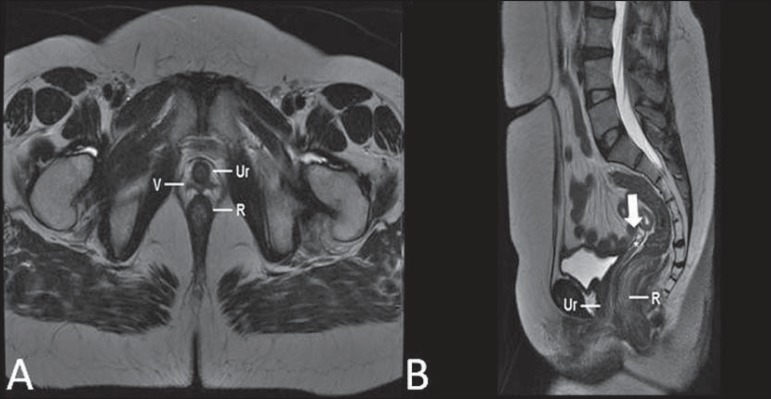
Turner syndrome. Multiplanar MRI T2-weighted sequence (**A,B**) demonstrate streak uterus and ovaries (arrow), short vagina (V) located between the rectum (R) and the urethra (Ur).

Turner syndrome (or 45,X) is the most common chromosomal sexual abnormality in women, and one of the main causes of primary amenorrhea. It is characterized by the absence of a copy of the X chromosome (45,X0), and is associated with hypertension, glucose intolerance, inflammatory bowel disease, hypothyroidism and gonadal dysgenesis. Typical MRI findings include streak uterus and ovaries, and short vagina^(4)^ (Figure 7).

### Gartner duct cyst

**Figure 8:**
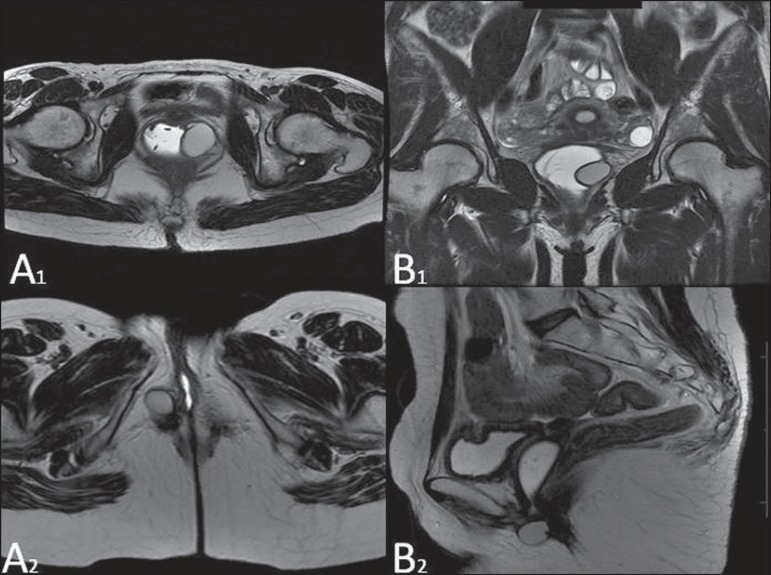
Gartner duct cyst – axial (**A1**) and sagittal (**B1**) MRI T2-weighted sequences demonstrating a cyst located in the left lateral vaginal wall, above the level of the pubic symphysis. Bartholin gland cyst – axial (**A2**) and sagittal (**B2**) T2-weighted sequences of another patient demonstrating cystic lesion outside the vaginal canal, on the distal posterior wall of the vagina at right.

Gartner duct cyst is related to incomplete involution of the vaginal portion of the mesonephric duct. Generally, such cysts are small and asymptomatic, however they may cause dyspareunia, interfere with obstetric delivery and associate with urogenital tract malformations. They are located in the anterolateral and upper walls of the vagina, above the pubic symphysis^(1,3)^ (Figure 8).

## BENIGN ACQUIRED ABNORMALITIES

### Bartholin gland cysts

Bartholin glands are derived from the urogenital sinus, secrete mucus and are located in the vaginal vestibule. Bartholin gland cysts develop due to duct obstruction and are located either at the same level or below the pubic symphysis. They are generally asymptomatic, but may require drainage due to infection or development of abscess^(1,6)^ (Figure 8).

### Skene glands cysts

**Figure 9:**
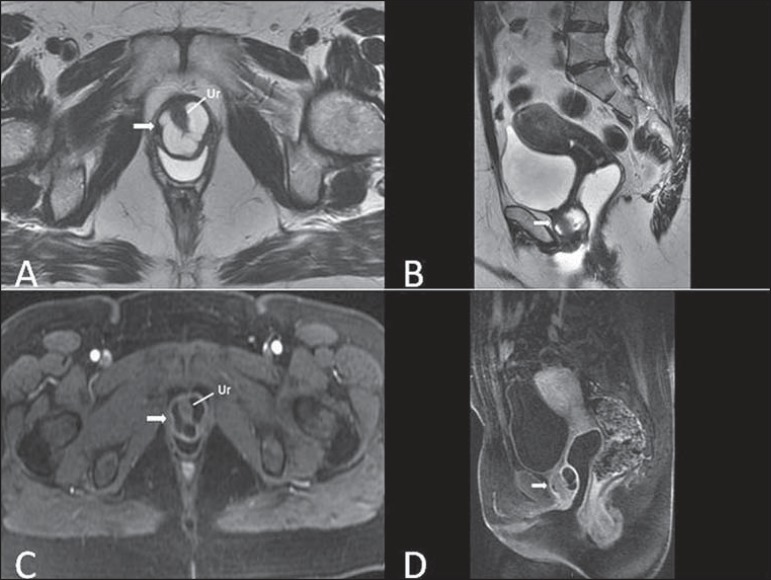
Cysts of the Skene glands. Multiplanar MRI T2-weighted (**A,B**) and contrast- enhanced T1-weighted (**C,D**) sequences identifying distal periurethral cysts (Ur) (arrows) located between the urethra and the vagina.

Skene glands are small periurethral glands located in the vaginal dome, adjacent to the inferior border of the dis-tal urethra and visible in cases of infection or obstruction. They are the equivalent to the male’s prostate and the main producers of PSA in women. Additionally, they are hormonedependent, increase in size during pregnancy and present atrophic in the climateric^(6,7)^ (Figure 9).

### Giant condyloma acuminatum

Giant condyloma acuminatum or Buschke-Loewenstein tumor of the perianal or anorectal regions is a rare entity.

**Figure 10:**
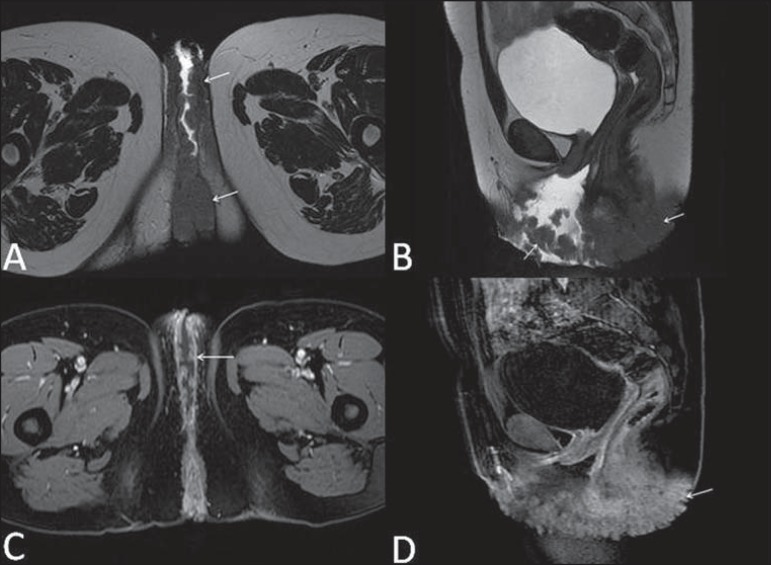
Giant condyloma acuminatum. Contrast-enhanced, multiplanar MRI T2- weighted (**A,B**) and T1-weighted (**C,D**) sequences of the pelvis demonstrating multiple cauliflower-like verrucous lesions in the anogenital region (arrows). After contrast medium injection, marked contrast uptake by the lesion was observed.

Generally, such lesions are large-sized and aggressive, prone to ulceration and infiltration into deeper tissues. They present high rate of recurrence (66%) as well as high rate of malignant transformation into squamous cell carcinoma (56%), but without distant metastases^(8)^ (Figure 10).

### Vaginal endometriosis

**Figure 11:**
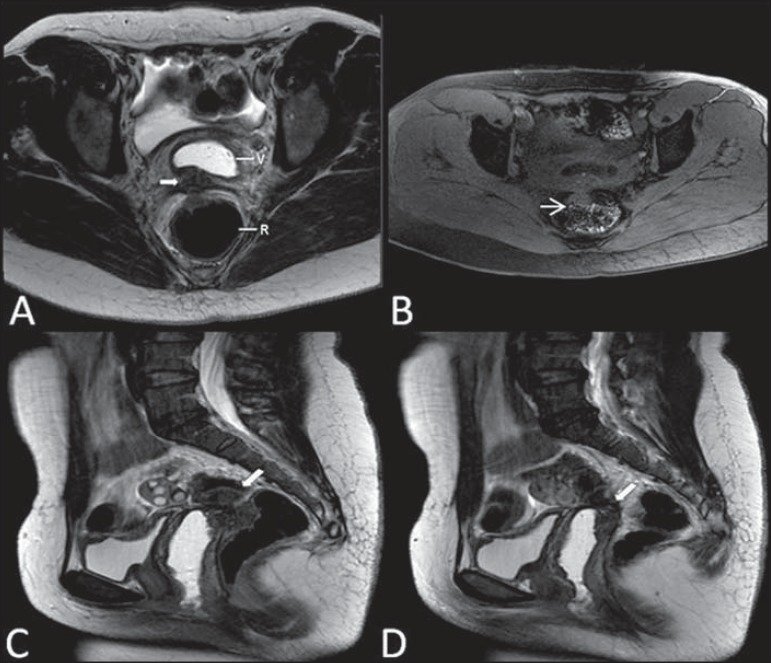
Multiplanar MRI T1-weighted (**B**) and T2- weighted (**A,C,D**) sequences of the pelvis demonstrating the presence of a focus of endometriosis with low signal intensity in the vaginal dome (bold arrows), with signs of local tissue retraction and extension to the anterior wall of the rectum, characterizing infiltrative endometriosis intermingled with a focus of high signal intensity corresponding to hemorrhagic focus (thin arrow). V, vagina; R rectum.

Endometriosis is defined by the presence of endometrial glands and stroma outside the uterine cavity. Frequently, it is found in pelvic fibromuscular structures such as uterosacral ligaments and ovaries. Vaginal location is frequent and may manifest with deep dyspareunia and dysmenorrhea^(9)^ (Figure 11).

## NEOPLASTIC DISEASES

### Primary neoplasms of the vagina

**Figure 12:**
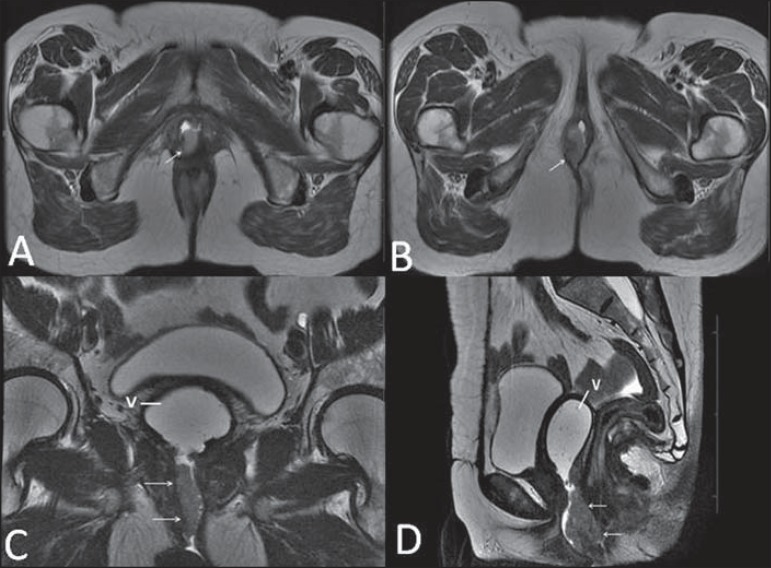
Spinocellular carcinoma. Axial (**A,B**), coronal (**C**) and sagittal (**D**) multiplanar MRI T2- weighted sequences showing the presence of a solid, lobulated mass in the posterior and right lower vaginal walls (V). The tumor infiltrates the rectovaginal fat plane (arrows).

**Figure 13:**
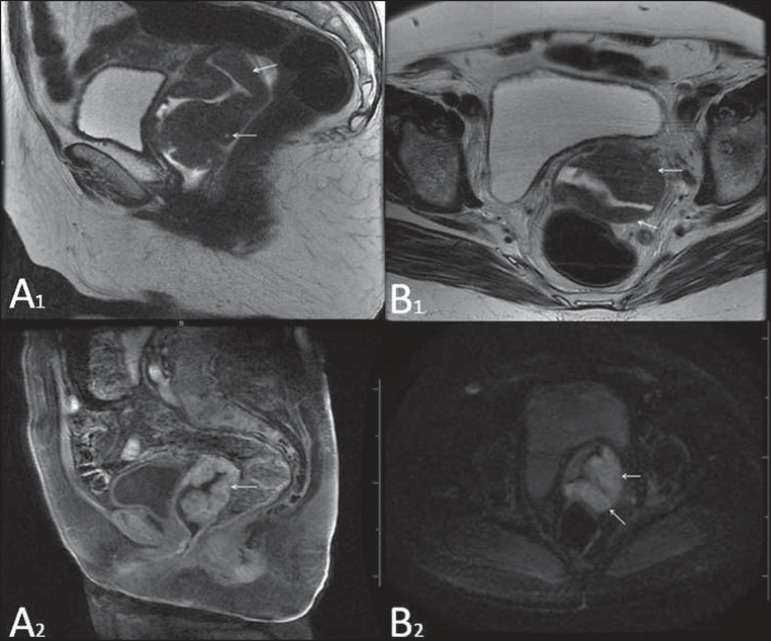
Vaginal melanoma. Sagittal (**A1**) and axial (**B1**) MRI T2-weighted sequences showing the presence of a lobulated mass with low signal intensity (arrows) affecting the anterior and posterior vaginal walls, extending throughout its entire length up to the vaginal ostiuml. MRI of another patient – contrast-enhanced T1-weighted sequence with fat saturation (**A2**) demonstrates a hypervascular lesion deeply invading the vagina. Diffusion-weighted image (**B2**) acquired with b = 750 s/mm^2^ shows significant diffusion restriction (arrows).

Primary vaginal neoplasms are rarely found. Spinocellular carcinoma represents approximately 85% of the primary malignant tumors of the vagina and develops from the posterosuperior vaginal wall (Figure 12). Other primary tumors are mainly adenocarcinoma, melanoma (Figure 13) and sarcomas^(10)^.

Staging: stage 0 – carcinoma *in situ*; stage I – tumor limited to the vaginal wall; stage II – tumor involving subvaginal tissue, without extension to the pelvic wall; stage IIItumor extending to the pelvic wall; stage IV – tumor extending to the true pelvis or involving the mucosa of the bladder or rectum; stage IV a – involvement of adjacent organs; IV b – involvement of distant organs.

### Secondary neoplasms of the vagina

**Figure 14:**
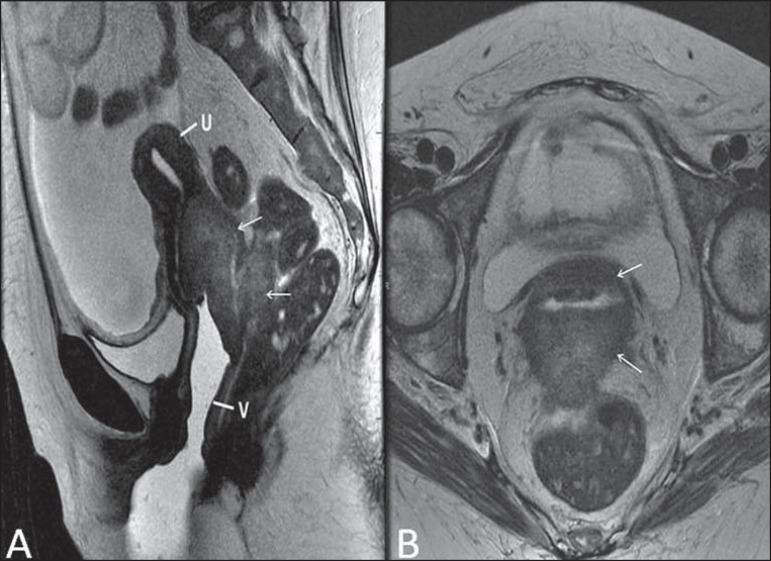
Uterine cervix adenocarcinoma with locally invasive tumor. Sagittal (**A**) and axial (**B**) MRI T2-weighted sequences demonstrating a heterogeneous and infiltrative lesion extending towards the vaginal dome, rectovaginal septum and mesorectal fascia (arrows). V, vagina; U, uterus.

**Figure 15:**
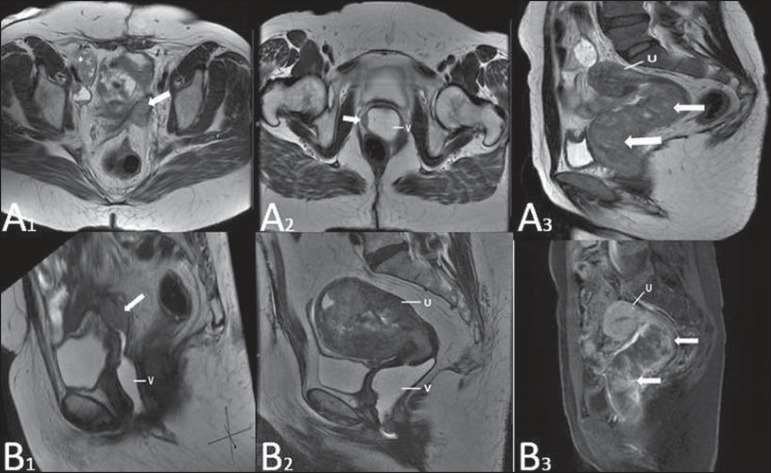
Vaginal metastasis from ovary adenocarcinoma – Axial (**A1**) and sagittal (**B1**) multiplanar MRI T2-weighted sequences demonstrate lymph node enlargement (asterisk) and peritoneal carcinomatosis, including an infiltrating lesion in the vaginal dome (arrows). V, vagina. Vaginal metastasis from endometrial carcinoma – Axial (**A2**) and sagittal (**B2**) MRI T2-weighted sequences demonstrating the primary tumor filling the endometrial cavity (U) and a well-defined nodule (skip lesions) with intermediate signal intensity in the right anterior wall of the vagina (arrow). V, vagina. Vaginal metastasis from uterine cervix squamous cell carcinoma – MRI T2-weighted (**A3**) and contrastenhanced T1-weighted (**B3**) sequences demonstrating ill-defined mass originating from the uterine cervix and extending towards the lower uterine segment and lower third of the vagina (arrows).

Secondary neoplasms of the vagina are more common than the primary ones and represent 80% of the vaginal tumors^(10)^, occurring by direct dissemination of tumors from adjacent pelvic organs (Figure 14), and rarely being of lymphatic or hematogenic origin. Most commonly, ovaries, endometrium, uterine cervix and rectum are sites of origin of such neoplasms (Figure 15).

## MISCELLANEA

### Vaginal prolapse

**Figure 16:**
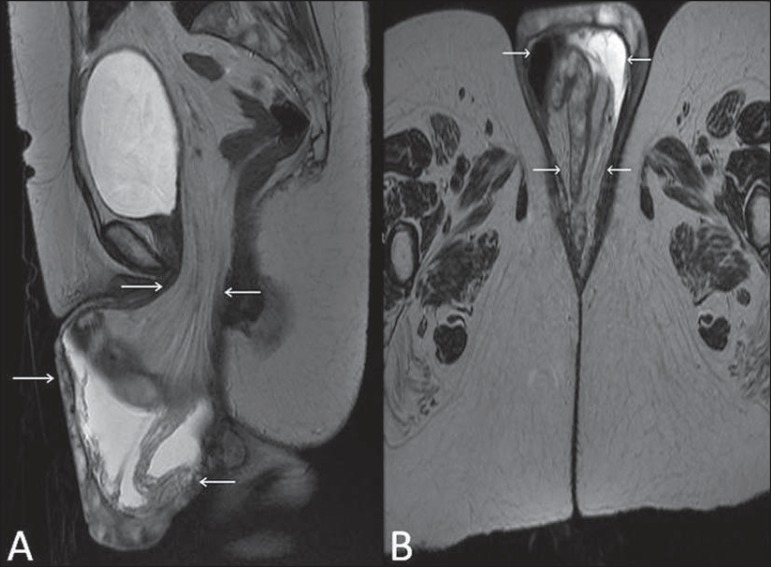
Pelvic floor prolapse. Sagittal (**A**) and dynamic axial (**B**) MRI T2-weighted sequences demonstrate large prolapse of the urogenital hiatus characterized by inversion of the vaginal dome, small bowel loops and abdominal fat protrusion.

It is a prevalent and debilitating symptom caused by the weakening of the pelvic floor and looseness of suspension structures. Main risk factors include multiparity, advanced age, menopause, obesity, conjunctival tissue diseases, smoking and chronic pulmonary obstructive disease. The symptoms are related to urinary and bowel incontinence, and sexual dysfunction^(11)^ (Figure 16).

### Post-radiotherapy vaginal fistulas

**Figure 17:**
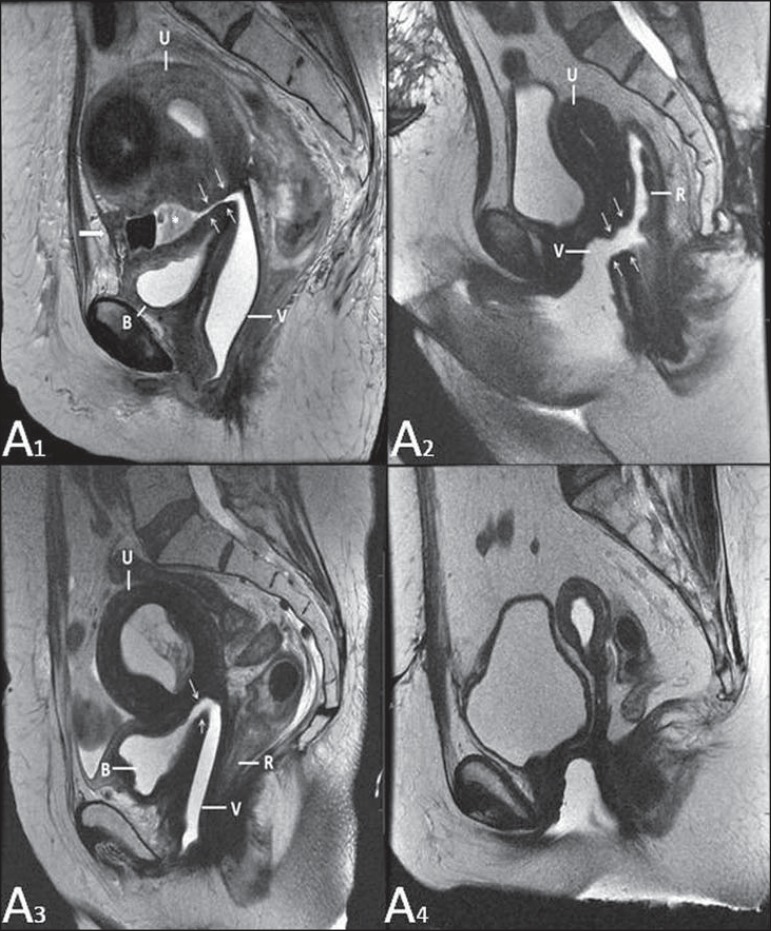
Post-radiotherapy complications. **A1:** Fistulous path (thin arrows) between the anterosuperior vaginal wall and the vesicouterine pouch. A small amount of heterogeneous fluid (asterisk) and anterior displacement of the peritoneal fold (bold arrow) are observed. V, vagina; U, uterus; B, bladder. **A2:** Fistulous path (arrows) between the rectum and the vagina. **A3:** Large vesicovaginal communication (arrows). Distension of uterine cavity determined by cervix stenosis (U). V, vagina; B, bladder; R, rectum. **A4** Stenosis of the upper third of the vagina, 10 months after radiotherapy (late complication).

Radiotherapy is widely utilized in the treatment of gynecological cancer, particularly in the case of uterine cervix cancer, and may trigger the development of fistulas, induce progressive obliterating endarteritis, resulting in mucosal surfaces necrosis/rupture. Approximately 2% of the patients submitted to radiotherapy for uterine cervix cancer develop fistulas that may occur up to 30 years after the treatment^(12)^ (Figure 17).

### Transexuality

**Figure 18:**
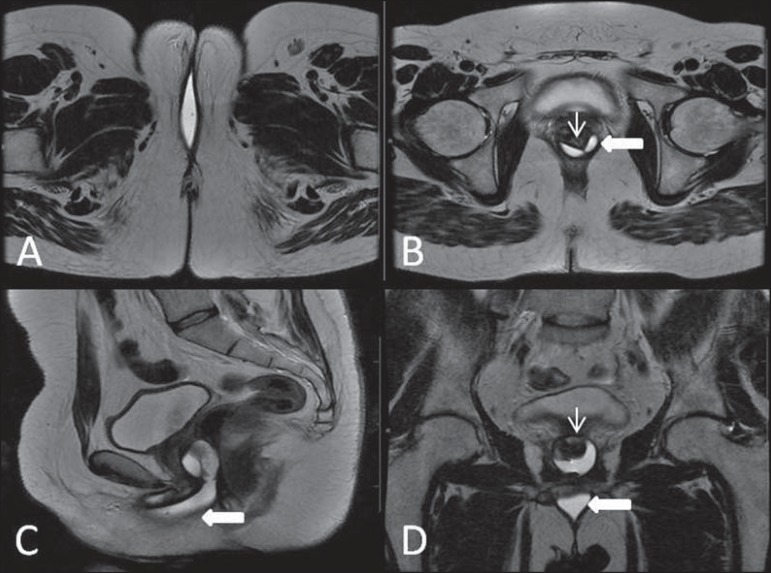
Surgical evaluation for maleto- female sex reassignment. Axial (**A,B**) sagittal (**C**) and coronal (**D**) MRI T2- weighted sequences demonstrate neovagina (bold arrows) and the remains of the corpora cavernosa and of the corpus spongiosum and urethra (thin arrows).

Sex reassignment surgeries have been performed for more than 30 years. The surgical procedure includes bilateral orchiectomy and penectomy and creation of urethrostomy, neovagina, labial structures and neoclitoris. MRI is the best imaging method to evaluate the pelvic anatomy in such patients^(13)^ (Figure 18).

## CONCLUSION

MRI is a very useful tool to evaluate the vagina and can provide essential data for diagnosis, therapeutic planning, detection of complications and follow-up. Thus, radiologists must be familiar with the scan protocols and with the data that must be reported for appropriate clinical decisions making.
